# Resistance of Garlic Cultivars to *Bradysia odoriphaga* and Its Correlation with Garlic Thiosulfinates

**DOI:** 10.1038/s41598-017-03617-9

**Published:** 2017-06-12

**Authors:** Guodong Zhu, Yin Luo, Ming Xue, Fangyuan Zhou, Haipeng Zhao, Guixia Ji, Fang Liu

**Affiliations:** 1 College of Plant Protection, Shandong Agricultural University, Key Laboratory of Biology of Vegetable Pests and Diseases, Shandong Province, 271018 China; 2 Non-point Source Pollution Remediation Laboratory, Ecology Institute, Shandong Academy of Sciences, Shandong Province, 250014 China

## Abstract

Garlic, a widely cultivated global vegetable crop, is threatened by the underground pest *Bradysia odoriphaga* in China. Previous reports indicated that garlic essential oil, of which the dominant components are sulfides or thiosulfinates, exhibits insecticidal activity against pests. However, it is unclear whether the resistance of garlic to *B. odoriphaga* is related to thiosulfinates. Here, we compared the resistance of 10 garlic cultivars at various growth stages to *B. odoriphaga* by field investigation and indoor life-table data collection. Furthermore, the relationship between thiosulfinates content and resistance, as well as the toxicity of garlic oil and allicin against *B. odoriphaga* larvae was determined. Field surveys demonstrated that the garlic cultivars Qixian and Cangshan possessed the highest resistance, while Siliuban and Yishui were the most sensitive. When reared on Qixian, *B. odoriphaga* larval survival and fecundity declined by 26.2% and 17.7% respectively, but the development time was prolonged by 2.8 d compared with Siliuban. A positive correlation was detected between thiosulfinates content and resistance. Furthermore, garlic oil and allicin exhibited strong insecticidal activity. We screened out 2 pest-resistant cultivars, for which thiosulfinate content was highest. Additionally, the insecticidal activity displayed by sulfides and allcin suggests their potential for exploitation as botanical insecticides.

## Introduction

Plant resistance to insects plays an important role in the long-term coevolution of insect phytophagy^1^. It relies on physical barriers, chemical defenses and induced direct or indirect plant defenses^2^. Phytochemicals play a vital role in the chemical defense against herbivores, which causes adverse impacts on the development and fecundity of insects alone or synergistically^3^. Recently, various phytochemicals such as glucosinolates, cyanogenic glucosides, alkaloids, phenolics and proteinase inhibitors have been proven to play obvious roles in plant resistance to insects^4, 5^. Extensive research on plant resistance, especially with regards to phytochemicals, provides new impetus for Integrated Pest Management (IPM)^6^.

Garlic, *Allium sativum* (Liliaceae: Allium), is an important commercial crop widely grown around the world, especially in Asia and North Africa^7^, and China is the largest global exporter of garlic^8^. It is also one of the most commonly used ingredients as a flavor enhancer for sausage. Additionally, garlic is appreciated for its medicinal properties, and various garlic preparations are widely used as health care products^9–11^. A number of studies showed that sulfur-containing organic compounds, such as thiosulfinates, played important role in these function^12^. The most important active substance, allicin (2-propene-1-sulfinothioic acid S-2-propenyl ester), formed during lysis of alliin by alliinase, as antibacterial, antiviral, antifungal and antiprotozoal properties, and also has beneficial effects on the cardiovascular and immune systems^12–15^. Clinical trials confirmed that the allicin extracted from garlic can inhibit the proliferation of cultured cancer cells^15^. In addition, allicin has been documented to reduce *Campylobacter jejuni* colonization in broilers when added to the drinking water^16^. Allicin was even found to be capable of inhibiting DNA and protein synthesis in *Salmonella enterica* and had an immediate effect on RNA synthesis^17^. More research is needed to determine the efficacy of allicin in various industrial areas.

Root maggot is a devastating soil pest that feeds on garlic bulbs. In China, where the technology of film mulched planting has been promoted, the chive maggot *Bradysia odoriphaga* Yang et Zhang (Diptera: Sciaridae) has become the most significant pest, replacing the onion fly *Delia antiqua*, which was the primary root maggot pest in the past^18^. *Bradysia odoriphaga* feeds on 7 plant families and more than 30 plants species, especially Chinese chive (*Allium tuberosum*.), garlic (*Allium sativum*), welsh onion (*Allium fistulosum*) and onion (*Allium cepa*)^19, 20^. *Bradysia odoriphaga* larvae tend to aggregate in fields and directly damage plants by feeding on the root and corm tissues in the growth medium, resulting in weak or withered plants. Since the larvae primarily damage the underground portions of plants, they are difficult to prevent or control. One of the most prevalent management practices against *B. odoriphaga* is the application of insecticides, such as organophosphates, carbamates and neonicotinoids^21^. However, insecticide use is increasingly restricted due to environmental pollution and human health concerns, as well as the development of pesticide resistance^22^. Therefore, breeding insect-resistant cultivars and seeking environmentally friendly insecticides are important channels for the effective control of pests. China is the largest cultivator of garlic and harbors abundant germplasms, including more than 370 cultivars^23^, providing the invaluable resources required for the exploitation and utilization of cultivars.

Previous research found that the development of *B. odoriphaga* was adversely affected when reared on garlic compared with Chinese chives; the most favorable host^24, 25^. A component analysis of the ethanol extract of liliaceous hosts showed that sulfides, the major components of the pungent odor of garlic^11, 26^, play an important role in host plant resistance to *B. odoriphaga* ^24^. Some reports confirmed that garlic extracts exhibited insecticidal activity against storage pests, such as *Tribolium castaneum* (Herbst)^27^ and *Sitotroga cerealella* (Lepidoptera: Gelechiidae)^28^. Diallyl disulfide and diallyl trisulfide, the stable polysulphide breakdown sulfides of allicin (an unstable thiosulfinate), are two major components in garlic essential oil^29–31^. Some studies investigated that thiosulfinates contents differed among garlic cultivars resulted the different sulphide in garlic^32^. However, whether the resistance of garlic cultivars to *B. odoriphaga* differs and could be related to thiosulfinates, or whether sulfides exhibit insecticidal activity against *B. odoriphaga*, has scarcely been studied.

In the current study, we investigated the differences in pest-resistance among garlic cultivars by field investigation. Then, we demonstrated life table for *B. odoriphaga* reared on 10 garlic cultivars using the age-stage, two-sex life table method, which is able to portray the actual dynamics of the population more accurately than the traditional life table by comprehensively analyzing the information between different developmental stages and different sexes^33, 34^. This work also investigated the correlation between garlic cultivar resistance to the root maggot and thiosulfinates content in garlic. Additionally, the toxicity of garlic essential and allicin against *B. odoriphaga* were determined.

## Results

### Quantity of *B. odoriphaga* occurring in the fields of different garlic cultivars

**Figure 1:**
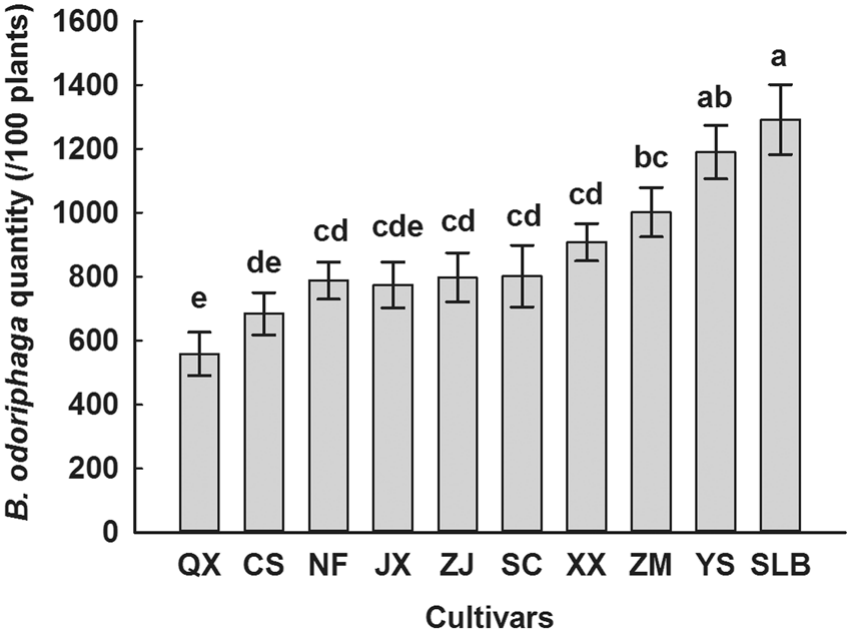
The quantity of *Bradysia odoriphaga* at different garlic cultivars in the field. The letter of QX, CS, NF, JX, ZJ, SC, XX, ZM, YS and SLB means the garlic cultivar of Qixian, Cangshan, Nanfang, Jinxiang, Zajiao, Sichuan, Xinxiang, Zhongmu, Yishui and Siliuban respectively. Values (quantity, mean ± s.e.) were analyzed with ANOVA, and different letters denoted significant differences by Tukey test (*P* < 0.05).

We recorded the degree of damage to the garlic bulbs caused by *B. odoriphaga* larvae for the different cultivars at the mature stage (May 20^th^, 2015). Figure 1 presents the significant differences in *B. odoriphaga* damage among the 10 cultivars (n = 50; df = 9, 40; F = 8.265; *P* < 0.001). The larval quantity on Siliuban and Yishui garlic cultivars was 1,302 and 1,194 larvae per 100 garlic plants respectively, constituting the most susceptible cultivars for root maggots. By contrast, the quantities of root maggots on Qixian and Cangshan cultivars were 558 and 684 larvae per 100 garlic plants respectively, constituting the most insect-resistant cultivars. The resistance level of the remaining cultivars was in between, with the quantity of root maggots recorded ranging between 774 and 1,002 larvae per 100 garlic plants.

### Life table of *B. odoriphaga* reared on 10 cultivars garlic at the mature stage

#### Development and reproduction

**Figure 2:**
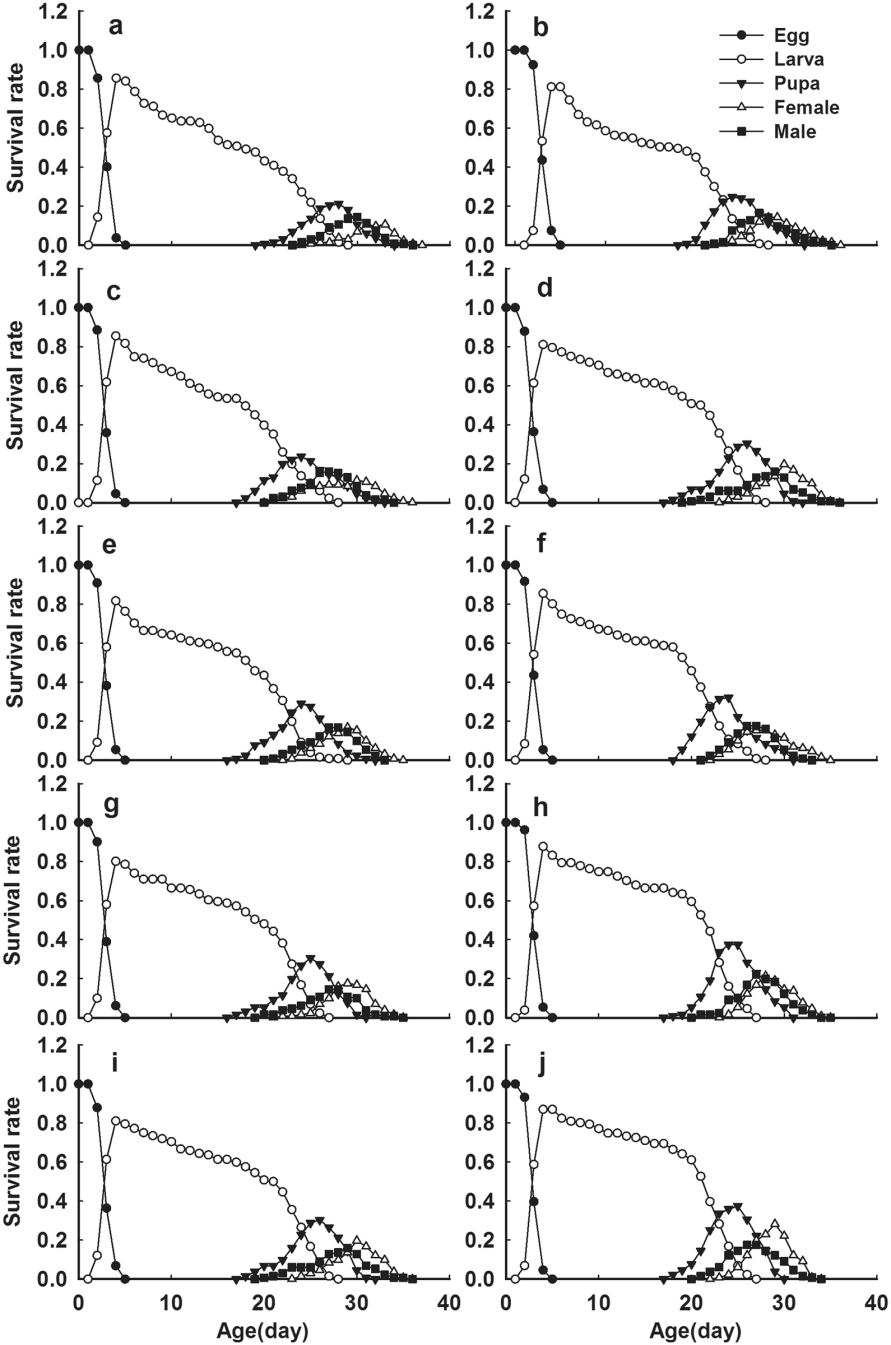
Age-stage-specific survival rate (*S*_*xj*_) of *B. odoriphaga* reared on different garlic cultivars, Qixian (**a**), Cangshan (**b**), Nanfang (**c**), Jinxiang (**d**), Zajiao (**e**), Sichuan (**f**), Xinxiang (**g**), Zhongmu (**h**), Yishui (**i**), and Siliuban (**j**).

*Bradysia odoriphaga* reared on the 10 garlic cultivars successfully reached the adult stage from eggs. Development, survival and fecundity parameters of *B. odoriphaga* reared on different garlic cultivars were noticeably different (Table 1). Obvious overlaps were observed in these curves (Fig. 2), showing the different development rates among individuals. The development time of larval stages for *B. odoriphaga* reared on the 10 cultivars was the most significantly different variable during the generation period. Particularly, we found that *B. odoriphaga* larvae grew slower when reared on the Qixian cultivar than on the others, and the larval developmental time was 22.29 d. The larval development times when reared on the Yishui (18.78 d), Zajiao (19.29 d), Sichuan (19.43 d), Nanfang (19.52 d), Zhongmu (19.84 d), Jinxiang (20.60 d) and Cangshan (21.03 d) cultivars were shorter than when reared on the Qixian cultivar. Additionally, the larval development time when reared on Siliuban cultivar was dramatically shorter than any of the others (18.71 d). There were no differences in the development times of the egg and pupa stages. The development time of eggs ranged between 3.25 d and 3.42 d across all 10 garlic cultivars, while the pupal stage was between 3.36 d and 3.49 d.

**Table 1:** Life tables of *Bradysia odoriphaga* on 10 cultivars of garlic.

Stages	Qixian	Cangshan	Nanfang	Jinxiang	Zajiao	Sichuan	Xinxiang	Zhongmu	Yishui	Siliuban
Egg(d)	3.26 ± 0.07 a	3.42 ± 0.07 a	3.25 ± 0.06 a	3.26 ± 0.02 a	3.31 ± 0.06 a	3.38 ± 0.06 a	3.31 ± 0.06 a	3.40 ± 0.06 a	3.28 ± 0.06 a	3.34 ± 0.06 a
Larva(d)	22.29 ± 0.28 a	21.03 ± 0.25 b	19.52 ± 0.33 d	20.60 ± 0.27 bc	19.29 ± 0.28 de	19.43 ± 0.26 de	19.82 ± 0.27 cd	19.84 ± 0.20 cd	18.78 ± 0.21 ef	18.71 ± 0.20 f
Pupa(d)	3.42 ± 0.11 a	3.43 ± 0.10 a	3.45 ± 0.09 a	3.48 ± 0.08 a	3.37 ± 0.09 a	3.49 ± 0.09 a	3.42 ± 0.08 a	3.48 ± 0.08 a	3.36 ± 0.07 a	3.49 ± 0.08 a
APOP(d)	1.65 ± 0.11 a	1.50 ± 0.10 ab	1.48 ± 0.09 ab	1.46 ± 0.09 ab	1.48 ± 0.09 ab	1.41 ± 0.08 b	1.49 ± 0.09 ab	1.54 ± 0.08 ab	1.46 ± 0.08 ab	1.30 ± 0.07 b
TPOP(d)	31.50 ± 0.47 a	30.29 ± 0.48 ab	28.75 ± 0.52 cd	29.83 ± 0.35 bc	28.79 ± 0.38 de	28.57 ± 0.41 de	29.09 ± 0.33 cd	28.93 ± 0.32 cd	26.80 ± 0.33 ef	27.53 ± 0.25 f
Female Longevity(d)	3.62 ± 0.10 b	3.81 ± 0.07 ab	3.73 ± 0.13 ab	3.81 ± 0.10 ab	3.76 ± 0.10 ab	3.78 ± 0.11 ab	3.71 ± 0.11 ab	3.67 ± 0.08 b	3.93 ± 0.10 a	3.73 ± 0.10 ab
Male Longevity(d)	3.96 ± 0.10 b	4.17 ± 0.07 a	4.39 ± 0.11 a	4.15 ± 0.06 ab	4.12 ± 0.07 ab	4.29 ± 0.11 a	4.10 ± 0.10 ab	4.34 ± 0.08 a	4.24 ± 0.08 a	4.22 ± 0.08 a
Larva Mortality (%)	56.06 ± 4.37 a	47.37 ± 4.33 ab	44.27 ± 4.30 ab	40.15 ± 4.35 bc	43.51 ± 4.42 bc	42.75 ± 4.28 bc	40.46 ± 4.16 bc	32.06 ± 4.02 cd	30.30 ± 3.94 d	29.77 ± 4.03 d
Fecundity (Egg/female)	93.71 ± 5.66 c	97.55 ± 4.81 bc	110.58 ± 4.26 ab	108.56 ± 4.55 ab	106.88 ± 4.50 bc	102.59 ± 3.40 bc	106.26 ± 4.77 bc	109.26 ± 4.11 ab	118.67 ± 3.93 a	113.81 ± 4.24 a

APOP, adult preovipositional stage; TPOP, total preovipositional stage (from egg to first oviposition). The values (mean ± s.e.) and standard errors were calculated using the bootstrap procedure with 10,000 bootstraps. The means followed by different letters in the same column are significantly different between cultivars at 5% significance level using the paired bootstrap test included in the computer program TWOSEX-MS Chart.

Differences in fecundity and adult longevity were observed across the different cultivars (Table 1). The mean fecundity of females was 93.71 eggs on Qixian cultivar, which was significantly lower than the other cultivars. The mean fecundity when reared on Cangshan cultivar was 96.27 eggs; the second lowest. Those reared on Yishui and Siliuban cultivars exhibited higher fecundity than on the other cultivars, with 113.81 and 118.67 eggs respectively. The fecundity when reared on other cultivars ranged between 102.59 and 110.58 eggs. With regards to Qixian cultivar, the adult longevity was observed to be the shortest at 3.62 d (females) and 3.96 d (males) respectively. Conversely, the longest adult longevity was observed on Yishui (3.93 d, females) and Nanfang cultivars (4.39 d, males). Male adults emerged earlier than female adults and had longer lifespans.

The *s*_*xj*_ curves indicate the probability that a newly hatched egg will survive to age *x* and develop to stage *j* (Fig. 2). The occurrence of death at the larval stage was an important factor causing the mortality differences of *B. odoriphaga* reared on different garlic cultivars (Table 1). Figure 2 suggests that the young larval stage is critical, being associated with a rapidly decreasing survival rate. The larval mortality of *B. odoriphaga* reared on Qixian cultivar was higher 56.06%, which was higher in comparison with those reared on the Cangshan, Nanfang, Xinxiang, Zajiao, Sichuan and Jinxiang cultivars (47.37%, 44.27%, 43.51%, 42.75% and 40.46% respectively). The mortality when reared on Zhongmu garlic and Yishui cultivars was lower at 32.06% and 30.30% respectively. The mortality when reared on Siliuban cultivar was the lowest at 29.77%. There were no significant differences between egg and pupal stage mortality (Supplementary Tables S1).

#### Population parameters

The population parameters were calculated using the bootstrap method presented in Table 2. The intrinsic rate of increase (r), the finite rate of increase (λ) and the net reproductive rate (R_0_) of *B. ordoriphaga* reared on Qixian and Cangshan cultivars were 0.082 d^−1^, 1.085 d^−1^, 14.91 d eggs and 0.097 d^−1^, 1.102 d^−1^, 21.24 d eggs respectively; significantly lower than the values obtained from other garlic cultivars. However, the values obtained from Siliuban (0.128, 1.137 and 41.67) and Yishui (0.124, 0.132 and 37.74) cultivars were higher than the others. Similarly, the mean generation time (T) obtained from Siliuban and Yishui cultivar (29.05 and 29.19 d) was shorter than that of Qixian cultivar (32.95 d). The population parameters of *B. ordoriphaga* reared on other cultivars were middle-ranked, such as an intrinsic rate of increase r = 0.109–0.118, finite rate of increase λ = 1.115–1.125 and the net reproductive rate R_0_ = 27.85–35.03.

**Table 2:** The effects of 10 cultivars of garlic on *Bradysia odoriphaga* population parameters.

Parameters	Qixian	Cangshan	Nanfang	Jinxiang	Zajiao	Sichuan	Xinxiang	Zhongmu	Yishui	Siliuban
Intrinsic rate of increase (r)	0.082 ± 0.007 e	0.097 ± 0.006 de	0.113 ± 0.006 bc	0.109 ± 0.005 cd	0.111 ± 0.006 bc	0.114 ± 0.005 bc	0.110 ± 0.005 cd	0.118 ± 0.005 ab	0.124 ± 0.005 ab	0.128 ± 0.004 a
Finite rate of increase (λ)	1.085 ± 0.007 e	1.102 ± 0.006 de	1.119 ± 0.007 bcd	1.115 ± 0.006 cd	1.117 ± 0.006 cd	1.121 ± 0.006 bc	1.116 ± 0.006 cd	1.125 ± 0.005 abc	1.132 ± 0.006 ab	1.137 ± 0.005 a
Net reproductive Rate (R_0_)	14.91 ± 3.09 d	21.24 ± 3.63 cd	27.85 ± 4.32 bc	29.61 ± 4.33 b	27.74 ± 4.26 bc	29.04 ± 4.12 bc	28.39 ± 4.32 bc	35.03 ± 4.67 ab	37.74 ± 4.97 ab	41.67 ± 5.04 a
Mean generation time (T)	32.95 ± 0.48 a	31.33 ± 0.53 b	29.75 ± 0.55 cd	31.15 ± 0.37 b	29.95 ± 0.39 cd	29.52 ± 0.39 cd	30.38 ± 0.36 bc	30.16 ± 0.31 c	29.19 ± 0.33 d	29.05 ± 0.27 d

The values (mean ± s.e.) and standard errors were calculated by using the bootstrap procedure with 10,000 bootstraps. The means followed by different letters in the same column are significantly different between cultivars at 5% significance level using the paired bootstrap test included in the computer program TWOSEX-MS Chart.

### Survival, fecundity and developmental time of *B. ordoriphaga* reared on garlic at different growth stages

**Figure 3:**
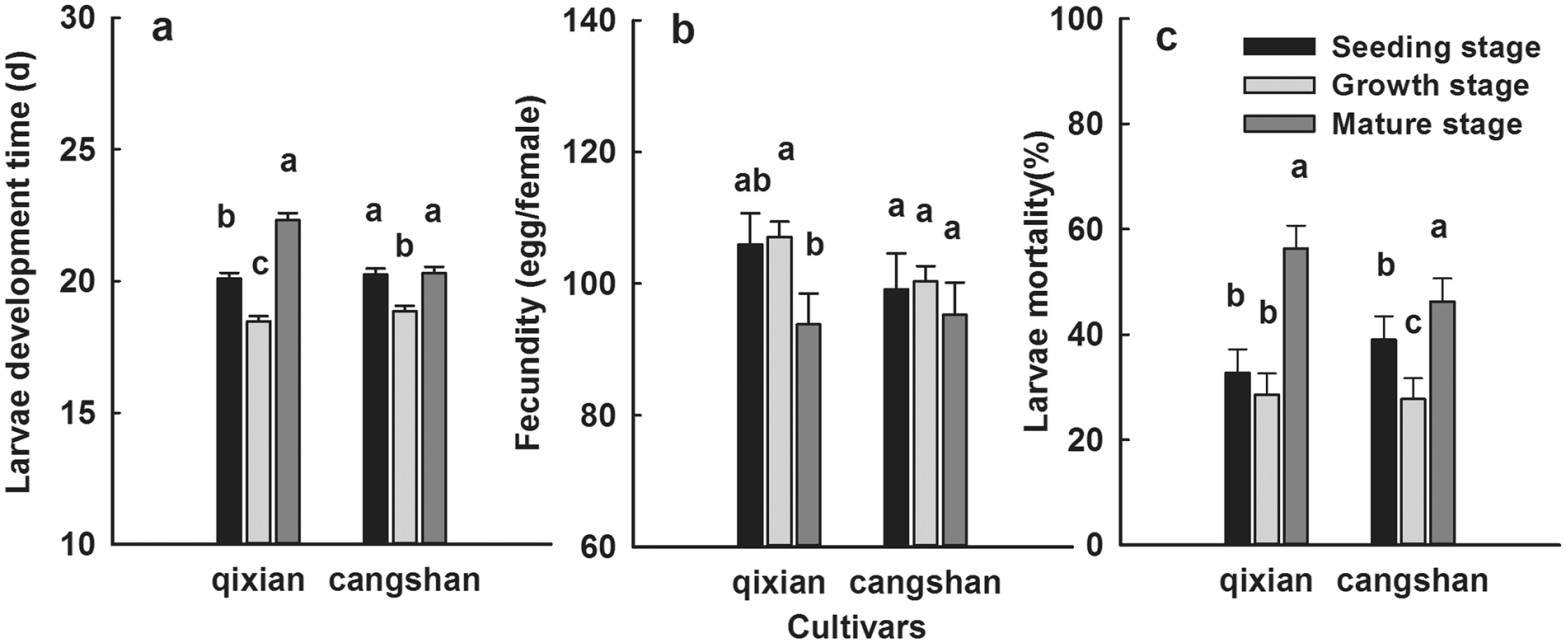
The larva development time (**a**), fecundity (**b**) and larva mortality (**c**) of *B. ordoriphyga* reared on two cultivars garlic in different growth stages. The values (mean ± s.e.) and standard errors were calculated by using the bootstrap procedure with 10,000 bootstraps. The columns covered by different letters are significantly different between growth stages of garlic at 5% significance level using the paired bootstrap test included in the computer program TWOSEX-MS Chart.

As for the larvae reared at different growth stages, the larval development time, fecundity and larval mortality of *B. ordoriphaga* differed significantly (Fig. 3). For example, for *B. ordoriphaga* reared on the Qixian cultivar we found that the larval development time at the mature stage (22.29 d) was dramatically longer than the seeding stage (20.02 days; *P* < 0.001) and growth stage (19.21d, *P* < 0.001), while the seeding and growth stage were significantly different (*P* < 0.001). The larval mortality at the mature stage (56.06%) was higher than at the seeding stage (38.21%; P < 0.001) and growth stage (29.30%; *P* < 0.001). The fecundity at the mature stage was 93.71 eggs for each female, which was significantly lower than the seeding stage (106.73 eggs for each female; *P* = 0.016) and growth stage (109.21 eggs for each female; *P* = 0.021). *B. odoriphaga* had a more adverse reaction when reared on the Qixian cultivar at the mature stage, followed by the seeding stage and growth stage, followed by the seeding stage and growth stage. These parameters showed a similar trend when *B. odoriphaga* was reared on the Cangshan cultivar.

### Correlation between thiosulfinates and insect resistance

#### Thiosulfinates content

The garlic were significantly different among the cultivars at the mature stage, and varied widely between the growth stages (Table 3). At the mature stage, the thiosulfinates contents in the Qixian, Cangshan and Nanfang cultivars were relatively high, at 30.95, 27.32 and 25.42 μmol/g respectively. Conversely, those in the Xinxiang, Sichuan, Zajiao, Jinxiang and Zhongmu cultivars were between 19.54 and 23.67 μmol/g, constituting in between values. However, the thiosulfinates contents in Yishui and Siliuban garlic were observed at a minimum level, at 18.27 and 17.90 μmol/g respectively. In addition, the thiosulfinates content at the mature stage in Qixian garlic was highest (30.95 μmol/g), followed by the seeding stage (23.75 μmol/g), while the lowest was recorded at the growth stage (19.90 μmol/g). Thiosulfinates contents in the other cultivars showed a trend similar to the Qixian cultivar, being the highest at the mature stage, at moderate levels in the seeding stage and the lowest in the growth stage, with the exception of the Sichuan, Zhongmu, Yishui and Siliuban varieties. The thiosulfinates contents of these 4 garlic cultivars exhibited no differences among the growth stages.

**Table 3:** Thiosulfinates contents in different vavieties garlic in different growth stages.

Cultivars	Seeding stage	Growth stage	Matural stage	df	F	P
Qixian	23.75 ± 0.90 ab, B	19.90 ± 1.22 abc, B	30.95 ± 1.06 a, A	(2, 6)	27.55	0.001
Cangshan	25.42 ± 0.66 a, A	21.82 ± 0.99 a, B	27.32 ± 0.69 ab, A	(2, 6)	12.41	0.007
Nanfang	23.62 ± 1.11 ab, AB	20.07 ± 0.85 abc, B	25.42 ± 1.23 b, A	(2, 6)	6.44	0.032
Jinxiang	19.81 ± 0.56 bcd, B	18.87 ± 0.65 abc, B	22.47 ± 0.47 bcd, A	(2, 6)	10.97	0.010
Zajiao	23.58 ± 0.83 ab, A	18.78 ± 0.76 abc, B	23.51 ± 1.25 bc, A	(2, 6)	8.06	0.020
Sichuan	23.02 ± 1.35 ab, A	21.57 ± 1.04 ab, A	23.67 ± 1.00 bc, A	(2, 6)	0.89	0.457
Xinxiang	21.35 ± 0.79 abc, AB	18.59 ± 0.55 abc, B	23.67 ± 0.78 bc, A	(2, 6)	12.67	0.007
Zhongmu	17.65 ± 0.81 bc, A	18.06 ± 0.76 abc, A	19.54 ± 1.48 cd, A	(2, 6)	0.86	0.468
Yishui	17.47 ± 0.60 bc, A	17.64 ± 0.58 bc, A	18.27 ± 0.3 6c, A	(2, 6)	0.65	0.554
Siliuban	16.88 ± 0.93 d, A	16.78 ± 0.62 c, A	17.90 ± 0.83 c, A	(2, 6)	0.60	0.579
df	(9, 20)	(9, 20)	(9, 20)			
F	12.30	3.86	17.34			
P	<0.001	0.006	<0.001			

Each value represents the average (±s.e.) of three replicates. The different small letters at the first column after each datum indicate significant differences in thiosulfinates contents among cultivars (*P* < 0.05) in the same growth stage, and the different capital letters at the second column after each datum indicate significant differences in thiosulfinates contents among growth stages (*P* < 0.05) in the same garlic cultivar. Data was analyzed with ANOVA (Tukey’s HSD).

#### Correlation between thiosulfinates and insect resistance

**Figure 4:**
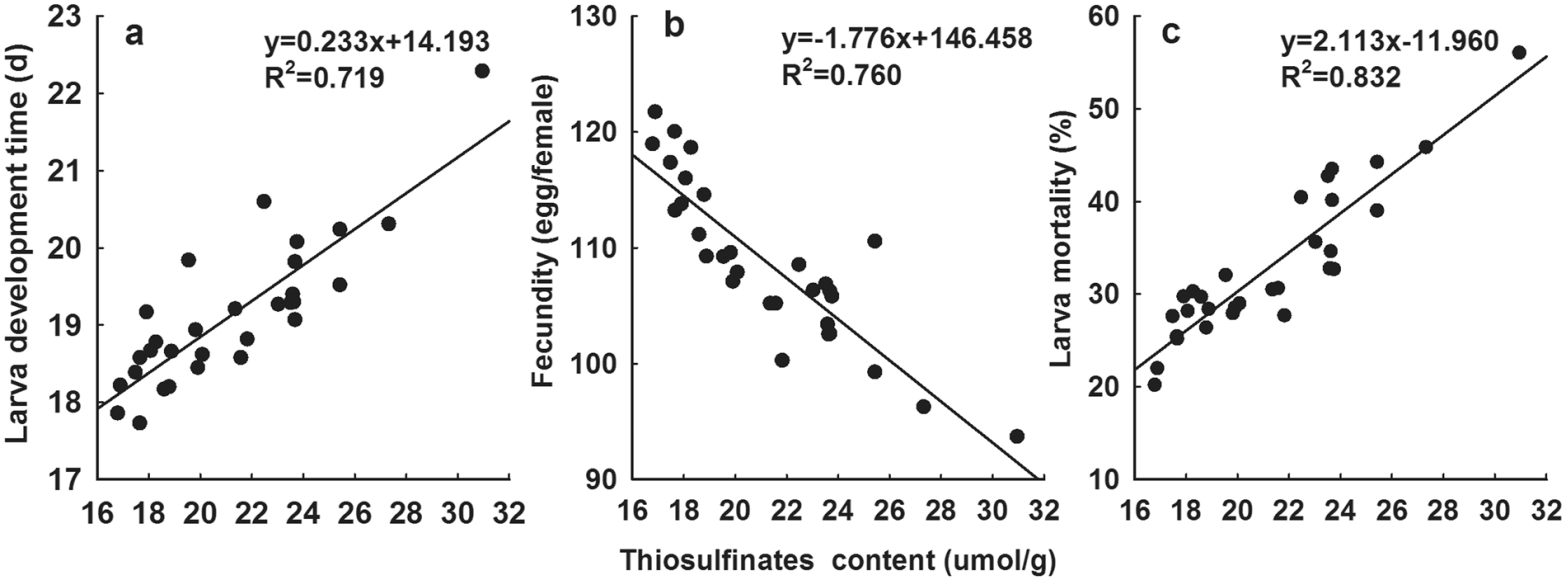
Correlation between thiosulfinates contents and insect-resistance among garlic cultivars. The correlation between thiosulfinates contents and larval development time (**a**), fecundity (**b**) and larval mortality (**c**) of *B. odoriphaga* reared on 10 garlic cultivars in different growth stages was analyzed by linear-regression analysis.

We analyzed the correlation between thiosulfinates content and the resistance performance to *B. ordoriphaga* (larval development time, fecundity and larval mortality) using linear-regression analysis. We found that thiosulfinates contents were significantly correlated with the insect-resistance of garlic to *B. ordoriphaga* (Fig. 4). Larval development time (df = 1, 28, F = 71.680, *P* < 0.001) and larval mortality (df = 1, 28; F = 139.135, P < 0.001) were positively linearly correlated with thiosulfinates content, while fecundity (df = 1, 28, F = 88.695, *P* < 0.001) showed a clear negative linear correlation. In other words, when thiosulfinates contents increased, larval development time was prolonged and the mortality increased, but fecundity decreased.

### Toxicity of synthetic garlic oil, allicin and 4 insecticides to the fourth instar of *B. odoriphaga*

The toxicity of the tested insecticides against the fourth instar developmental stage of *B. odoriphaga* is provided in Table 4. In terms of rapid effect (treated for 48 h), the botanical insecticides (synthetic garlic oil and allicin) showed unremarkable insecticidal activity when compared with Phoxim (LC_50_ = 1.751 mg/L) and Emamectin benzoate (LC_50_ = 40.594 mg/L). Synthetic garlic oil (LC_50_ = 65.574 mg/L) showed stronger insecticidal activity. In terms of chronic effects (treated for 96 h), synthetic garlic oil (LC_50_ = 24.706 mg/L) showed the most potent insecticidal activity, followed by allicin (LC_50_ = 50.372 mg/L). The toxicities of the garlic compounds were all determined to stronger than Sophocarpidine (LC_50_ = 86.925 mg/L) and Azadirachtin (LC_50_ = 72.079 mg/L).

**Table 4:** Toxicity of synthetic garlic oil, diallyl disulphide and 4 insecticides against fourth instar of *Bradysia odoriphaga*.

Insecticide	Treated for 48 h			Treated for 96 h		
	Regression equation y = ax + b	LC_50_ (95%CL) (mg/L)	(χ^2^, Sig)	Regression equation y = ax + b	LC_50_ (95%CL) (mg/L)	(χ^2^, Sig)
Phoxim	−3.213x + 0.615	1.751 (1.396~2.101)	(6.483, 0.999)	−3.215x + 0.710	1.663 (1.426~1.972)	(7.096, 0.998)
Synthetic garlic oil	−2.109x + 3.775	65.574 (49.787~81.760)	(3.713, 0.997)	−2.688x + 3.744	24.706 (20.260~29.455)	(7.489, 0.998)
Allicin	−2.509x + 5.159	114.189 (86.624~149.433)	(9.374, 0.995)	−3.182x + 5.416	50.372 (43.377~59.388)	(16.448, 0.959)
Emamectin benzoate	−4.543x + 7.307	40.594 (35.957~45.837)	(8.361, 0.998)	−4.643x + 7.306	37.445 (33.205~42.196)	(7.947, 0.998)
Azadirachtin	−2.613x + 5.648	144.949 (120.680~179.570)	(8.719, 0.997)	−3.434x + 6.379	72.079 (62.193~83.324)	(10.257, 0.990)
Sophocarpidine	−2.588x + 5.706	160.317 (132.714~201.137)	(8.123, 0.998)	−3.160x + 6.127	86.925 (74.521~101.646)	(9.153, 0.995)

The survival rate was recorded after 48 h and 96 h for the rapid and chronic effect respectively.

## Discussion

Three-to-four generations of *B. odoriphaga* per year are produced in the garlic fields of Shandong province in China. April to June is suitable for its growth, resulting in severe damage during the mature stage of garlic development^18^. In this study, the resistance of 10 garlic cultivars to *B. odoriphaga* was investigated for the first time. The result showed that the Qixian and Cangshan garlic cultivars exhibited the highest resistance to *B. odoriphaga*, while Yishui and Siliuban were the most susceptible (Fig. 1). Wang^23^ also reported that the Zhongmu, Cangshan and Jinxiang cultivars showed obvious resistance to *Delia antiqua*, however, we found that Zhongmu garlic was sensitive to *B. odoriphaga*. These differences may be due to regional variations or difference in planting conditions.

In general, the stronger the resistance of plant to a pest is, the longer the developmental duration of the insect lasts, resulting in lower survival and fecundity^35^. Previous research has indicated that although *B. odoriphaga* possesses a wide range of hosts, the different hosts exert various effects on *B. odoriphaga* ^24, 25^. Xue^24^ found that the survival and fecundity of *B. odoriphaga* declined when reared on garlic compared with Chinese chives and other liliaceous vegetables. In this study, our results revealed that the population growth rate of *B. odoriphaga* was influenced differently by the various garlic cultivars as observed by the differences in larval developmental time, larval mortality and fecundity, and consequently, the differences in the population parameters (r, R_0_, and T). When reared on the insect-resistant cultivars Qixian and Cangshan, the larval development time of *B. odoriphaga* was prolonged and the survival and fecundity decreased compared with those reared on insect-susceptible cultivars Siliuban and Yishui (Table 1); exhibiting major differences in resistance performance. Our findings are in agreement with many studies on the effects of host plants on the development, survival and reproduction of insects^35, 36^. The larval mortality was highest across the entire larval lifecycle, especially during the young stage (Fig. 2). This suggested that larvae in the young stage were the most vulnerable to external negative factors. Thus, this represents the ideal stage for controlling the insect population. Meanwhile, the different growth stages of garlic were found to differ significantly with respect to *B. odoriphaga* resistance, and the results indicated that garlic at the mature stage exhibited the highest resistance followed by the seeding stage, while the growth stage was the lowest (Fig. 3). Therefore, we recommend Qixian and Cangshan garlic as *B. odoriphaga-*resistant cultivars, and cultivation on a large scale might efficiently reduce the costs of *B. odoriphaga* control; a hypothesis which we verified under field conditions. The various effects on the development and fecundity of phytophagous insects resulting from host plants may also be attributed to nutrition and phytochemical differences in the different host plants^37^. Thus, an analysis of the sap composition of the garlic cultivars will help to clarify the factors affecting the population growth parameters of *B. odoriphaga*. Xue^24^ reported that *B. odoriphaga* reared on garlic cloves possessed lower survival and fecundity compared with those reared on garlic sprouts, and speculated that the differences in nutrition and phytochemicals led to this result.

Phytochemicals play an important role in plant insect-resistance^6, 38^. Xue^24^ reported that sulfides constituted the critical component for the resistance of *Allium* plants against *B. odoriphaga*. Wang^23^ analyzed the differences in resistance to *D. antiqua* among 34 garlic cultivars in the field, and found that the cultivar with high allicin content exhibited strong resistance. In addition, allicin accounted for about 60–90% of thiosulfinates in garlic^11, 26^. Our biochemical measurements confirmed that the thiosulfinates content in garlic was significantly correlated with resistance to *B. odoriphaga*. The cultivar with higher thiosulfinate content possessed higher insect-resistance, which confirms the results of Wang^23^. The difference in thiosulfinates content among various growth stages can also explain the differences in resistance mentioned above. This correlation may result from the insecticidal activity of sulfides or garlic essential oil as reported in previous studies.

Many plant essential oils possess potential insecticidal activity^39, 40^. As the majority of these chemicals degrade rapidly and have little or no harmful effects on the environment or humans^41, 42^, some of them including Azadirachtin and Sophocarpidine have been applied as insecticides. Garlic essential oil is known to possess repellent and insecticidal activity against arthropod pests^43–46^. Prowse^45^ also found that garlic oil possessed insecticidal efficacy against 2 target dipteran pests, *Delia radicum* (L.) and *Musca domestica* (L.). Additionally, garlic oil showed significant acaricidal effects, and exhibited lethal effects on *Tetranychus urticae* (Tetranychidae), reducing the fecundity of the treated mites^46^. Results in this study confirmed that garlic essential oil and allicin possessed good insecticidal activity against *B. odoriphaga* larvae, and their LC_50_ toxicity values were lower than that of the botanical insecticides Azadirachtin and Matrine. Additionally, allicin, an unstable thiosulfinate, decomposed into various sulphides easily at normal condition, and diallyl disulphide is the main breakdown products. Sulfides are known to be the dominant component of garlic oil extractions, comprising a mixture of diallyl disulfide, diallyl trisulfide, diallyl sulphide and other sulfides^11^. Our laboratory found that sulfides accounted for 99.9% of the garlic essential oil, while diallyl disulfide, diallyl sulfide and diallyl trisulfide accounted for 68.59%, 20.18% and 11.22% respectively. Elucidation of the pesticidal mechanism of garlic essential oil and their constituents is helpful in providing necessary information regarding the exploitation of the most appropriate formulation and delivery means. Moreover, according to the results of Yang^27^, diallyl trisulfide acts as a fumigant against the stored product pest *Tribolium castaneum* (Herbst). Our results confirmed that allicin exhibited a little low insecticidal activity against *B. odoriphaga* larvae compared with the garlic oil. We also speculated that sulphides, the decomposition products of thiosulfinate possess better insecticidal activity, and which sulphide, diallyl disulphide or diallyl trisulfide, playing dominant role needed further study.

Planting insect-resistant cultivars may reduce damage from pests; however, this method does not eliminate all damage. To ensure crop quality and yield, insecticides are frequently used in the field. Although many plant essential oils are known to possess insecticidal or repellent activity against insect pests, the control of *B. odoriphaga* to date has depended on synthetic chemicals. We have now confirmed that garlic essential oil exhibits strong insecticidal activity against *B. ordoriphaga*. Furthermore, Park^43^ discovered that garlic essential oil possessed high insecticidal activity against *Lycoriella ingenua* (Diptera: Sciaridae), a proximal species of *B. ordoriphaga*, and diallyl disulfide exhibited fumigant activity. These results all indicate that garlic essential oil or sulfides have the potential to act as novel botanical insecticides against dipteran pests. In addition, we also found that *B. odoriphaga* adults had positive taxis to low allicin concentration and phobotaxis to high concentration^47^. Thus, the insecticidal activity may be related to garlic oil concentration. Moreover, many studies have reported the expense of using botanical insecticides for pest control compared with chemical insecticides, as larger doses are required due to their weak potency. We found that a mixture of botanical insecticide (Azadirachtin) and chemical insecticide (Clothianidin) could control pests at a lower cost^48^. However, it is not clear whether garlic essential oil could be mixed with chemical insecticide for the control of *B. odoriphaga*. Further work needs to be conducted to confirm the dosage and methods of garlic essential oil in the control of *B. odoriphaga*.

## Methods

### Garlic cultivars

Ten garlic cultivars, namely Qixian (originally from Qixian, Henan), Cangshanpuke (originally from Cangshan, Shandong), Zajiao (originally from Laiwu, Shandong), Xinxiang (originally from Xinxiang, Henan), Jinxiang (originally from Jinxiang, Shandong), Sichuan (originally from Chengdu, Sichuan), Yishui (originally from Linyi, Shandong), Nanfang (originally from Guiyang, Guizhou), Zhongmu (originally from Zhongmu, Henan) and Siliuban (originally from Cangshan, Shandong), were obtained from the College of Horticulture, Shandong Agricultural University (Tai’an, China). These 10 garlic cultivars comprise the main cultivated cultivars, and many previous studies had have investigated their diversity^23, 32, 49, 50^. All garlic cultivars used in this study were planted in the experimental field of Shandong Agricultural University on October 5^th^ 2014, and harvested on June 5^th^ 2015. Before covering with plastic film, 330 g/L of the herbicide pendimethalin (EC) was applied. The 10 cultivars were planted randomly, and the planting area of every cultivar measured approximately 120 m^2^ (length 20 m; width 6 m) with 5 duplicates. During planting, the plants were sufficiently watered and no insecticides were applied.

### *B. odoriphaga* colonies

*Bradysia odoriphaga* colonies were originally obtained from a Chinese chive field in Tai’an, Shangdong, China in April 2013. Insect colonies were maintained in the Shandong Provincial Key Laboratory of Applied Microbiology and reared on an artificial diet for more than 10 generations. According to the rearing method described by Xue^24^, eggs, larvae and pupae were reared in culture dishes (Φ = 9 cm) covered with wet filter paper, and newly emerged adults were placed in pairs in individual oviposition containers (plastic cups of 3 cm diameter × 1.5 cm height) containing a piece of 30 mm filter paper moistened with deionized water. To facilitate egg collection, the artificial diet was cut into 1 cm-long pieces and placed in the rearing cages to allow females to oviposit. The *B. odoriphaga* colonies were maintained in growth cabinets at 25 ± 1 °C, 12 h: 12 h L: D, 75% ± 5% RH.

### Insecticides and synthetic garlic oil

Sophocarpidine (5% technical concentrate), emamectin benzoate (88.3%), azadirachtin (5%) and phoxim (95.5%) were supplied by the Key Laboratory of Pesticide Toxicology and Application Technique, Shandong Agricultural University, Tai’an. Alternatively, synthetic garlic oil (EO; containing 3 major constituents, 68.59% of diallyl disulfide, 11.22% of diallyl trisulfide, and 20.18% of diallyl sulphide: Supplementary Tables S2 and Fig. S1) and allicin (95.8%, CFN90201) were purchased from the Wuhan ChemFaces Limited. L-cysteine and 5,5′-dithiobis (2-nitrobenzoic acid; DTNB) were purchased from Sigma-Aldrich. Acetone and Tween 80 were purchased from Tianjin Caratton Industry Limited.

### Investigation of the occurrence of *B. odoriphaga* in the garlic field

Using a chess board sampling technique, on May 20^th^ 2015 we randomly selected 10 points in every cultivar of the garlic field with every 1 point being represented by 5 plants, and recorded the number of *B. odoriphaga* larvae on every garlic bulb.

### Life table study

The garlic plants exhibiting no injuries across the different growth stages (the seeding stage, November 2014; the growth stage, April 2015; and the mature stage, May 2015) were used as test plants. The *B. odoriphaga* colonies were reared on the garlic bulbs. Prior to the life table study, *B. odoriphaga* individuals were reared on these 10 garlic cultivars for a single generation. The eggs spawned by the adults reared on the different cultivars were gathered as the test insects. A total of 150 eggs (15 eggs from each female) were used for the life table study with each garlic cultivar. These eggs and hatched larvae were reared on the same cultivar sequentially. Every garlic cultivar bulb was cut into thin slices (approx. 3 mm) and placed in a separate petri dish. The eggs mentioned above were placed around the garlic thin slices. The eggs were observed daily and the hatching rates were recorded. Each day, newly hatched larvae were transferred into a new culture dish separately for supply of one of the diets. The survival of *B. odoriphaga* was recorded daily and fresh supplies of the diets were provided to avoid fungal growth until all adults perished. Deionized water was replenished daily to keep the filter paper moist. Just after the transformation from larvae into pupae, the pupae were moved to new petri dishes. After the emergence of adults, male and female insects were paired and placed in individual oviposition plastic containers. Adults were checked daily and the number of eggs of each individual was recorded until death.

### Physiological determination of thiosulfinate content in garlic

The garlic cloves were ground with a mortar and mixed with water (25 mL per 0.5 g). The suspension was shaken for 15 min at 25 °C and filtered through gauze. The undissolved material was removed by centrifugation at 300 × g for 4 min. The thiosulfinate contents were determined using the method described by Han in 1995^26^. The reaction mixture included 0.5 mL garlic extract and 4.5 mL cysteine solution (200 μM, Hepes buffer pH 7.5). After 10 min, every 1 mL reaction mixture was incubated in a cuvette with 4 mL DTNB (1.5 mM, phosphate buffer pH 7.5). The residual concentration of cysteine in the mixture was determined by measuring the amount of 2-nitro-5-thiobenzoate (NTB) formed after reaction with DTNB and the molar extinction coefficient (1 cm light path) of 14,150 at 412 nm.

1 $$\mathrm{Total}\,\,{\rm{thiosulfinate}}\,{\rm{content}}=\frac{{\rm{\Delta }}\mathrm{A412}\times \mathrm{2500}}{2\times \mathrm{14150}}\times 1000\,({\rm{\mu }}\mathrm{mol}/g)$$

### Bioassays

Bioassays were conducted on the newly emerged fourth instar larvae of *B. odoriphaga* using a standard contact and stomach bioassay method (insecticide-impregnated filter method). Two pieces of filter paper (Φ = 9 cm) were immersed into the tested solution and then one was flattened in the culture dish. Fresh Chinese chive cauloids (0.5 cm) were cut and dipped into the test solution for 10 s with gentle agitation, and air dried at room temperature. Twenty *B. odoriphaga* larvae were placed around the Chinese chive cauloid placed on the filter paper, and the remaining piece of filter paper was placed on top of the tested larvae. Every treatment included 100 larvae for 5 replications, using a pure water treatment as the control. Serial dilutions (mg/L) of the active ingredient diluted with 0.1% Tween 80 solution were prepared. The test larvae were reared on the Chinese chive pseudo stems mentioned above and maintained in growth cabinets at 25 ± 1 °C, 12 h: 12 h L:D, 75% ± 5% r. h. Larval survival was checked and recorded following treatment for 48 h or 96 h.

### Data analysis

Raw data of all individuals in the life table were analyzed according to the age-stage two-sex life table theory^33^. The developmental time, longevity, fecundity and population parameters and their mean values and standard errors were estimated using the bootstrap method included in the computer program TWOSEX-MS Chart^33, 34^. Differences in *B. odoriphaga* populations among the cultivars were compared using the paired test included in the software.

We tested the other variables for homogeneity of group variances using Levene’s test and normality using the Kolmogorov-Smirnov test prior to statistical analysis. For the analysis, the quantity of *B. odoriphaga* on each garlic cultivar was regarded as the dependent variable, while the garlic cultivars were regarded as the independent variables in one-way ANOVA followed by Tukey’s HSD multiple comparisons. With regards to the thiosulfinates content analysis at the same growth stages, the content in each garlic cultivar was regarded as the dependent variable, while the garlic cultivars were regarded as independent variables using one-way ANOVA followed by Tukey’s HSD multiple comparisons. Conversely, when analyzing the thiosulfinates content in the same garlic cultivar, the content at each growth stage was regarded as the dependent variable, while the growth stages were regarded as independent variables in the above-mentioned method. In the correlation analysis, thiosulfinates content in each cultivar and each growth stage of garlic was regarded as the independent variable, and *B. odoriphaga* larval development time, mortality, and fecundity reared on each cultivar were regarded as the dependent variables in the regression analysis followed by linear regression. In the bioassays, the number of dead *B. odoriphaga* larvae when treated with each insecticide dose (including the 0 mg kg^−1^ group, i.e., the control group) was regarded as the response frequency, while the doses were regarded as the covariate, and the total tested number of larvae in each treatment was regarded as the total observed in the regression analysis, which was followed by Probit. All analyses were performed with PASW Statistics 18.0.0 (2009; SPSS Inc. Quarry Bay, HK). Figures were constructed using SigmaPlot 12.0.

## Electronic supplementary material

Supplementary
