# Supplementary material for: Resistance of Garlic Cultivars to Bradysia odoriphaga and Its Correlation with Garlic Thiosulfinates
Source: Sci Rep. 2017 Jun 12;7:3249. doi: 10.1038/s41598-017-03617-9 (PMC5468350; doi:10.1038/s41598-017-03617-9)
Supplement: Supplementary file 1 — Supplementary [file 41598_2017_3617_MOESM1_ESM.doc]

# **Resistance of Garlic Cultivars to *Bradysia odoriphaga* and Its Correlation with Garlic Thiosulfinates**

Guodong Zhu^1^, Yin Luo^1^, Ming Xue^1*^, Fangyuan Zhou^2^, Haipeng Zhao^1^, Guixia Ji^1^, Fang Liu^1^

^1^ College of Plant Protection, Shandong Agricultural University, Key Laboratory of Biology of Vegetable Pests and Diseases, Shandong Province, 271018, China.

^2^ Non-point Source Pollution Remediation Laboratory, Ecology Institute, Shandong Academy of Sciences, Shandong Province, 250014, China.

* Correspondence: *E-mail:* [xueming@sdau.edu.cn](mailto:xueming@sdau.edu.cn)

**Supplementary:**

# **Figures and tables for “Resistance of Garlic Cultivars to *Bradysia odoriphaga* and Its Correlation with Garlic Thiosulfinates”**

**Table S1 Mortality of egg, pupa, female and male of** ***Bradysia odoriphaga* on 10 cultivars of garlic in life table study**.

| **Stages** | **Qixian** | **Cangshan** | **Nanfang** | **Jinxiang** | **Zajiao** | **Sichuan** | **Xinxiang** | **Zhongmu** | **Yishui** | **Siliuban** |
| --- | --- | --- | --- | --- | --- | --- | --- | --- | --- | --- |
| **Egg**  **Mortality (%)** | 5.30±1.94 a | 6.02±1.99 a | 4.58±1.78 a | 5.30±2.07 a | 4.58±1.91 a | 4.58±1.84 a | 4.58±1.81 a | 4.58±1.76 a | 5.30±1.93 a | 4.58±1.85 a |
| **Pupa**  **Mortality** **(%)** | 2.27±1.29 a | 1.52±1.05 a | 2.29±1.33 a | 2.27±1.28 a | 2.29±1.29 a | 1.53±1.10 a | 3.05±1.49 a | 2.29±1.31 a | 3.79±1.64 a | 1.53±1.04 a |
| **Female**  **Mortality (%)** | 15.91±3.17 c | 21.78±3.57 c | 25.19±3.63 bc | 27.27±3.93 ab | 26.72±3.92 bc | 25.95±3.82 ab | 28.24±3.83 ab | 32.06±4.15 ab | 31.82±4.11 a | 36.64±4.31 a |
| **Male**  **Mortality (%)** | 20.45±3.58 b | 23.30±3.67 ab | 23.66±3.63 ab | 25.00±3.70 ab | 22.90±3.65 ab | 25.19±3.89 ab | 23.66±3.69 ab | 29.01±3.93 a | 28.79±3.95 a | 27.48±4.12 a |

The values (mean±s.e.) and standard errors were calculated using the bootstrap procedure with 10,000 bootstraps. The means followed by different letters in the same column are significantly different between cultivars at 5% significance level using the paired bootstrap test included in the computer program TWOSEX-MS Chart.

.


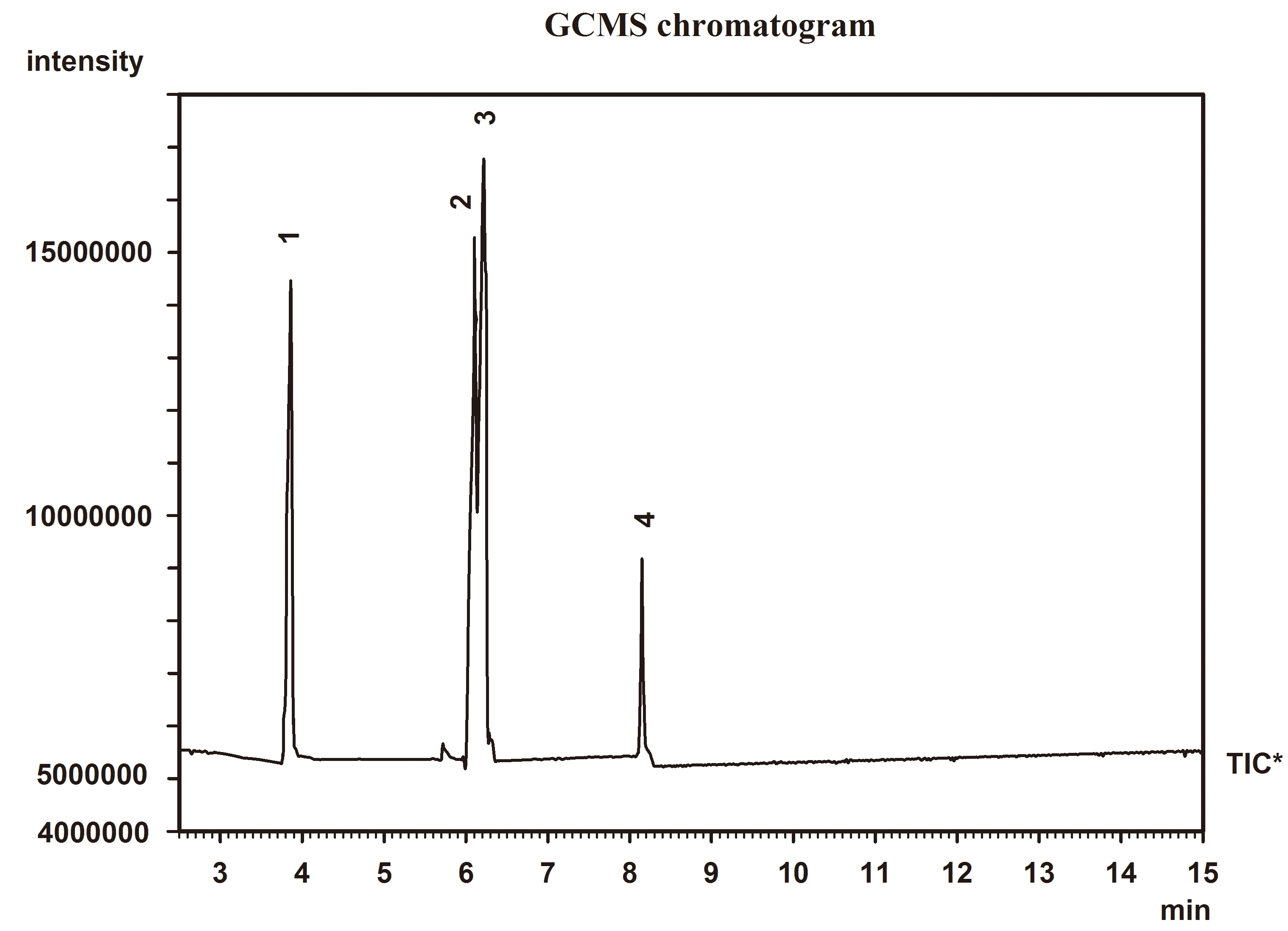


**Figure S1** Gas chromatogram of chemical composition of synthetic garlic oil

**Table S2 Gas chromatogram of chemical composition of synthetic garlic oil**

| Peak Number | Composition | Content (%) | Molecular formula | Molecular weight |
| --- | --- | --- | --- | --- |
| 1 | Diallyl sulfide | 20.18 | C_6_H_10_S | 114 |
| 2 | Diallyl disulfide | 19.57 | C_6_H_10_S_2_ | 146 |
| 3 | Diallyl disulfide | 49.02 | C_6_H_10_S_2_ | 146 |
| 4 | Diallyl trisulfide | 11.22 | C_6_H_10_S_3_ | 178 |
| 0 | Others | 0.001 |  |  |
